# Lysosomal Exoglycosidase Profile and Secretory Function in the Salivary Glands of Rats with Streptozotocin-Induced Diabetes

**DOI:** 10.1155/2017/9850398

**Published:** 2017-12-31

**Authors:** Mateusz Maciejczyk, Agnieszka Kossakowska, Julita Szulimowska, Anna Klimiuk, Małgorzata Knaś, Halina Car, Wiesława Niklińska, Jerzy Robert Ładny, Adrian Chabowski, Anna Zalewska

**Affiliations:** ^1^Department of Physiology, Medical University of Bialystok, 2c Mickiewicza Street, 15-233 Bialystok, Poland; ^2^Department of Conservative Dentistry, Medical University of Bialystok, 24a M. Sklodowskiej-Curie Street, 15-274 Bialystok, Poland; ^3^Department of Pedodontics, Medical University of Bialystok, 24a M. Sklodowskiej-Curie Street, 15-274 Bialystok, Poland; ^4^Department of Cosmetology, Lomza State University of Applied Sciences, Akademicka 1 str, 18-400 Lomza, Poland; ^5^Department of Experimental Pharmacology, Medical University of Bialystok, 37 Szpitalna Street, 15-767 Bialystok, Poland; ^6^Department of Histology and Embryology, Medical University of Bialystok, 13 Waszyngtona Street, Bialystok, Poland; ^7^Department of Emergency Medicine and Disasters, Medical University of Bialystok, 37 Szpitalna Street, 15-767 Bialystok, Poland

## Abstract

Before this study, there had been no research evaluating the relationship between a lysosomal exoglycosidase profile and secretory function in the salivary glands of rats with streptozotocin- (STZ-) induced type 1 diabetes. In our work, rats were divided into 4 groups of 8 animals each: control groups (C2, C4) and diabetic groups (STZ2, STZ4). The secretory function of salivary glands—nonstimulated and stimulated salivary flow, *α*-amylase, total protein—and salivary exoglycosidase activities—N-acetyl-*β*-hexosaminidase (HEX, HEX A, and HEX B), *β*-glucuronidase, *α*-fucosidase, *β*-galactosidase, and *α*-mannosidase—was estimated both in the parotid and submandibular glands of STZ-diabetic and control rats. The study has demonstrated that the activity of most salivary exoglycosidases is significantly higher in the parotid and submandibular glands of STZ-diabetic rats as compared to the healthy controls and that it increases as the disease progresses. Reduced secretory function of diabetic salivary glands was also observed. A significant inverse correlation between HEX B, *α*-amylase activity, and stimulated salivary flow in diabetic parotid gland has also been shown. Summarizing, STZ-induced diabetes leads to a change in the lysosomal exoglycosidase profile and reduced function of the salivary glands.

## 1. Introduction

Diabetes mellitus (DM) is a group of frequent metabolic disorders characterized by abnormalities in insulin secretion and/or insulin action [1]. The primary clinical manifestation of type 1 diabetes (DM1; OMIM %222,100) is an elevated blood glucose level, which leads to chronic hyperglycemia and subsequent acute and chronic complications, including micro- and macrovascular disease. Metabolic abnormalities in DM1 disturb the function of numerous human organs and systems, including also the salivary glands, which influences oral cavity homeostasis [2, 3]. Many studies have shown that DM1 is strongly associated with oral fungal and bacterial infections, changes in the composition and buffering properties of saliva, higher incidence of lichen planus, and dental caries, as well as periodontal disease [2–4]. However, the pathogenesis of these oral complications is still not fully understood in the context of DM1.

The oral cavity is an integral part of the entire human body. It is commonly known that maintenance of the oral homeostasis depends largely on salivary glycoproteins suspended in an aqueous solution of saliva, as well as on the glycoconjugates which are integral parts of the salivary gland structures [5, 6]. Salivary glycoproteins (e.g., immunoglobulins, lactoferrin, and salivary peroxidase system) and glycolipids participate in the interaction between salivary proteins, carbohydrates, oral bacteria, and viruses and thereby play an important role in the oral immune defense mechanisms [7]. Salivary glycoconjugates such as mucins (MUC1, MUC4, MUC5B, MUC7, and MUC19), proline-rich glycoproteins (PRGs), and kallikrein may also ensure the appropriate hydration of the oral mucosa and maintain the proper pH of the stimulated and nonstimulated saliva [8, 9].

Salivary glands produce the intracellular lysosomal enzymes that hydrolysis the oligosaccharide chains of salivary glycoconjugates. This group, known as salivary lysosomal exoglycosidases, includes N-acetyl-*β*-hexosaminidase (HEX and NAG) and its isoenzymes A (HEX A) and B (HEX B), *β*-glucuronidase (GLU), *α*-fucosidase (FUC), *β*-galactosidase (GAL), and *α*-mannosidase (MAN) [10]. HEX, the most active salivary exoglycosidase, hydrolyses N-acetylglucosamine (GlcNAc) or N-acetylgalactosamine (GalNAc) from the nonreducing ends of salivary glycoconjugates, whereas salivary GLU hydrolyses the *β*-glycosidic bonds from the *β*-glucuronides [10, 11]. Recently, more and more attention has been paid to the determination of salivary lysosomal glycosidases in many oral and systemic diseases [11]. Bierc et al. [12] noted a significant increase in HEX, GLU, and GAL activity in the salivary gland tumor tissue, while Waszkiewicz et al. [13] reported that HEX determination may be a useful marker for salivary dysfunction caused by a single dose of ethanol. Determination of lysosomal exoglycosidases also appears to have a significant diagnostic value in screening and monitoring chronic periodontitis [14], cancer [12, 15], asthma [16], alcohol dependence [17, 18], Lyme borreliosis [19], and rheumatoid arthritis [19, 20], as well as type 1 and type 2 diabetes mellitus [21–23]. It has been demonstrated that increased activity of lysosomal hydrolases in saliva reflects their elevated synthesis/release, which may be associated with lysosomal membrane damage [22, 24]. A positive correlation between the activity of salivary exoglycosidases and the degree of salivary gland dysfunction has also been reported [20, 25]. However, there is no data concerning the lysosomal exoglycosidase profile in the submandibular/parotid gland and/or saliva of DM1 patients as well as in appropriate animal models. Accordingly, the aim of this study was to evaluate the lysosomal exoglycosidase profile and their relationship with salivary amylase activity and salivary flow rate in the salivary glands of rats with streptozotocin- (STZ-) induced type 1 diabetes.

## 2. Materials and Methods

The study was approved by the Local Committee on the Ethical Use of Animals, Medical University of Bialystok, Poland (permission number 29/2014).

### 2.1. Animals

Healthy male Wistar rats (6–8 weeks) were obtained from the Department of Experimental Pharmacology, Medical University of Bialystok, Poland. Rats weighed between 180 and 210 g. Animals were housed in a controlled environment (20-21°C ± 2°C, 12 h light/12 h dark) and had free access to conventional food (standard laboratory rat chow Agropol, Motycz, Poland) and drinking water. Five days after their arrival, the rats were divided randomly into two main groups [control (C) and streptozotocin- (STZ-) induced diabetes (STZ) group] of 16 animals each (*n* = 16). Then, also at random, each group was divided into two equal subgroups (*n* = 8), depending on the number of weeks [two (C2, STZ2) and four (C4, STZ4) weeks] counted from the moment of the diabetes induction until the sacrifice of rats.

Diabetes was induced by intraperitoneal (i.p.) injection of STZ (60 mg/kg BW in 0.1 M citrate buffer, pH 4.5; Sigma, St. Louis, MO, USA) by a qualified staff member. Animals of the control group received only the 0.1 M citrate buffer. 48 hours after STZ administration, diabetes condition was measured with a glucometer (Accu-Check Active, Roche, France) and rats with blood glucose level above 250 mg/dL were considered as diabetic. After 2 (C2, STZ2) and 4 (C4, STZ4) weeks of the STZ injection, rats were weighed and anesthetized using phenobarbital (80 mg/kg BW, i.p.).

To measure the rate of salivary secretion, sleeping rats were placed on a heating couch (37°C, slope of 30°). Nonstimulated salivary flow rate was estimated by a qualified staff member by using a preweighed sterile cotton swab. The swab was placed at the bottom of the oral cavity of every rat for 15 minutes, and the volume of the resulting saliva was calculated by subtracting the weight of the dry cotton swab from the weight of the salivated swab after 15 minutes. It was assumed that 1 *μ*L of saliva weighs 1 mg. The salivary secretion was stimulated by intraperitoneal injection of pilocarpine hydrochloride (5 mg/kg BW; Sigma, St. Louis, MO, USA). The measurement of the stimulated salivary secretion was performed similarly to the measurement of the unstimulated salivary flow 5 minutes after the injection of pilocarpine. The measurement lasted 5 minutes [26]. Next, the parotid and submandibular glands were taken by a qualified laboratory staff member immediately after the collection of blood samples from the abdominal aorta. Tissues were placed on ice and purified from blood elements and fat, and then the material was frozen in liquid nitrogen and stored at −80°C until use. Plasma insulin levels were determined by the enzyme-linked immunosorbent assay (ELISA) (Rat Insulin ELISA kit, Shibayagi Co., Japan) in duplicate samples.

### 2.2. Preparation of Homogenates

Salivary glands were thawed and rinsed in ice-cold phosphate buffered saline (PBS; 0.02 M, pH 7.0). To determine the activity of HEX, HEX A, HEX B, GLU, GAL, MAN, and FUC, tissues were weighed (laboratory balance KERN PLI 510-3 M), minced, and diluted 1 : 9 (*w*/*v*) in 0.15 M KCl with 0.2% Triton X-100 (POCH, Gliwice, Poland). To determine the total protein concentration, tissues were diluted in ice-cold PBS (1 : 9; *w*/*v*). To determine the salivary amylase activity, salivary glands were diluted in ice-cold phosphate buffer of pH 6.9 (1 : 9; *w*/*v*). In order to prevent sample oxidation and proteolysis, butylated hydroxytoluene (BHT, Sigma-Aldrich, Germany; 10 *μ*L 0.5 M BHT in acetonitrile (ACN)/1 mL PBS) and proteolysis inhibitor (1 tablet/10 mL PBS; Complete Mini Roche, France) were added [27, 28]. After that, tissues were homogenized on ice with a glass homogenizer (5000 rpm for 1 min; Omni TH, Omni International, Kennesaw, GA, USA). The resulting suspensions were sonicated on ice with an ultrasonic cell disrupter (1800 J per sample, 3 times for 20 s each; UP 400S, Hielscher, Teltow, Germany). The homogenates were centrifuged at 3000 ×g for 20 min in 4°C (MPW 351, MPW Med. Instruments, Warsaw, Poland) to collect the supernatant and be assayed immediately.

### 2.3. Biochemical Determinations

The performed analysis covered the activity of HEX (EC 3.2.1.52), its isoenzymes HEX A and HEX B, GLU (EC 3.2.1.31), GAL (EC 3.2.1.23), MAN (EC 3.2.1.24), FUC (EC 3.2.1.51), and salivary amylase (EC 3.2.1.1) as well as total protein concentration.

The activity of lysosomal exoglycosidases was estimated colorimetrically by the method of Marciniak et al. [29] (HEX, HEX A, and HEX B) and Chojnowska et al. [10] (GLU, GAL, MAN, and FUC). As a substrate for determining the activity of HEX, HEX A, and HEX B, 4-nitrophenyl-N-acetyl-*β*-glucosaminide (Sigma, St. Louis, MO, USA) was used. 4-nitrophenyl-*β*-D-glucuronide, 4-nitrophenyl-N-acetyl-*β*-D-galactopyranoside, 4-nitrophenyl-N-acetyl-*α*-D-mannopyranoside, and 4-nitrophenyl-N-acetyl-*α*-D-fucopyranoside (Sigma, St. Louis, MO, USA) were used to evaluate the activity of GLU, GAL, MAN, FUC, respectively. HEX isoenzymes were determined after the heat denaturation of the isoform A. HEX A activity was calculated from the difference between the activity of HEX and HEX B. The intensity of the yellow color of the released 4-nitrophenol (PNP) was measured at 405 nm using Infinite M200 PRO Multimode Microplate Reader, Tecan. All analyses were performed in triplicate samples. The results were converted into 100 mg of the total protein.

The activity of salivary amylase was determined colorimetrically by the method of Fisher and Stein [30] with 3,5-dinitrosalicylic acid (POCH, Gliwice, Poland) as a substrate reaction. A standard curve was made for maltose (POCH, Gliwice, Poland) in a concentration range of 0–180 mg/mL. All analyses were performed in triplicates. The results were converted into 100 mg of the total protein.

Total protein content was measured using bicinchoninic acid method (BCA) with bovine serum albumin (BSA) as a standard (Thermo Scientific PIERCE BCA Protein Assay Kit, Rockford, IL, USA). All analyses were performed in duplicates.

### 2.4. Histological Examination

The right parotid and submandibular glands were embedded in paraffin for making five-micron sections. The sections were deparaffinized, rehydrated, and stained with Hematoxylin and Eosin (H&E) and then examined by a qualified histologist under a light microscope (OPLYMPUS BX 51, OLYMPUS) using a graticule 40 and 60x magnification.

### 2.5. Statistical Analysis

Statistical analysis was performed using the Statistica 10.0 system (Statsoft, Cracow, Poland) according to the Mann–Whitney test. The data were presented as median, minimum, and maximum. To evaluate the relationship between two quantitative variables, nonparametric Spearman correlation was used. The statistical significance was defined as *p* ≤ 0.05.

## 3. Results

### 3.1. Characteristics of Rats

The body weight of the control rats was significantly higher in week 4 of the experiment as compared to week 2. The weight of rats with STZ-induced diabetes was significantly lower as compared to the control rats both in week 2 and week 4 of the experiment. The glucose concentration in the whole blood of rats with STZ-induced diabetes was significantly higher in both week 2 and week 4 of the study as compared to the control rats. The glucose concentration in STZ-diabetic rats was significantly higher in week 4 as compared to week 2 of the experiment. In the group of rats with STZ-induced diabetes, plasma insulin concentrations were below the minimum detection level in both week 2 and week 4 of the study (Table 1).

The parotid gland weight of rats with STZ-induced diabetes in week 4 of the study was significantly lower as compared to the control rats. The weight of the parotid gland in the group of STZ-diabetic rats was significantly lower in week 4 as compared to week 2 of the experiment (Table 1).

### 3.2. Salivary Flow and Total Protein Content

In the 4th week of the experiment, unstimulated salivary secretion was significantly lower in the group of diabetic rats compared to the controls. Stimulated salivary flow was considerably lower both in the 2nd and 4th week of the experiment in the group of rats with STZ-induced diabetes compared to the respective controls (Table 2).

In the 4th week of the study, the concentration of total protein was significantly lower in parotid glands of rats with diabetes compared to healthy controls (Table 2).

### 3.3. HEX

In parotid glands of STZ-diabetic rats, HEX-specific activity in week 4 of the experiment was significantly higher compared to HEX activity in week 2. We observed significantly lower HEX-specific activity in submandibular glands of rats with 4-week-old diabetes compared to rats with 2-week-old diabetes, and considerably higher HEX activity in diabetic rats in week 2 compared to the controls (C2 group).

**Figure 1:**
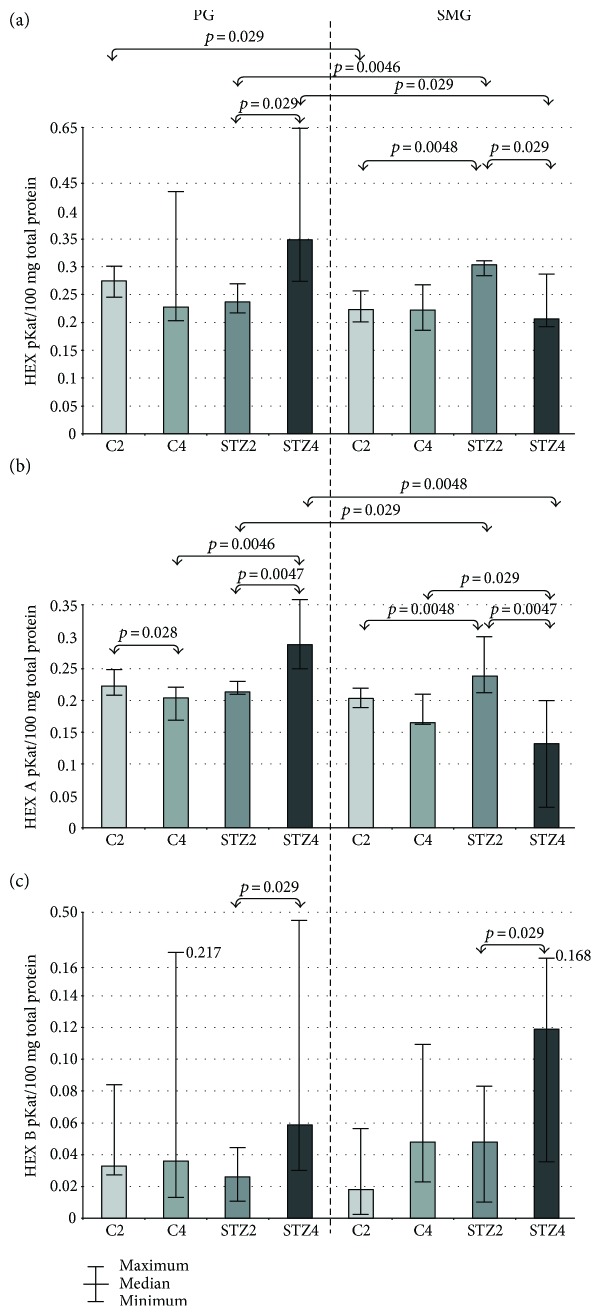
The effect of STZ-induced diabetes on activity of HEX, HEX A, and HEX B in the parotid and submandibular glands of rats. C2 and C4 are the control groups; HEX: N-acetyl-*β*-hexosaminidase; HEX A: N-acetyl-*β*-hexosaminidase A; HEX B: N-acetyl-*β*-hexosaminidase B; PG: parotid glands; SMG: submandibular glands; STZ2 and STZ4 are the diabetic groups.

The specific activity of HEX in parotid glands of the control rats in the 2nd week of the experiment was significantly higher compared to submandibular glands of these rats, whereas in the group of diabetic rats in the 2nd week of the experiment, it was considerably lower in parotid glands than in submandibular glands. In week 4 of the experiment, HEX-specific activity in diabetic rats was significantly higher in parotid glands compared to submandibular glands (Figure 1(a)).

### 3.4. HEX A and HEX B

In parotid glands of the control rats, HEX A-specific activity was significantly lower in week 4 of the experiment compared to week 2, while in the rats with experimentally induced type 1 diabetes, it was considerably higher in week 4 compared to week 2. The specific activity of HEX A in parotid glands of STZ-diabetic rats in week 4 was significantly higher than in the controls. We have demonstrated significantly lower HEX A-specific activity in submandibular glands of rats with 4-week-old diabetes compared to rats with 2-week-old diabetes. In the submandibular glands of diabetic rats, HEX A-specific activity was considerably higher in the 2nd week of the experiment, but significantly lower in the 4th week compared to the respective controls.

We have found that HEX A-specific activity in rats with experimentally induced type 1 diabetes was significantly lower in the 2nd week of the study, and in the 4th week, it was considerably higher in parotid glands of these rats compared to their submandibular glands (Figure 1(b)).

Both in parotid and submandibular glands of rats with diabetes, the specific activity of HEX isoenzyme B (HEX B) was significantly higher in week 4 of the experiment compared to week 2 (Figure 1(c)).

### 3.5. GAL

GAL-specific activity in parotid glands of the control rats as well as STZ-induced diabetic rats was significantly higher in the 4th week of the experiment compared to the 2nd week. In both the 2nd and 4th week of the study, we demonstrated considerably higher GAL-specific activity in parotid glands of diabetic rats compared to healthy controls. In submandibular glands of the control rats, GAL-specific activity in the 4th week was significantly higher in comparison to the 2nd week of the experiment. In submandibular glands of rats with diabetes, GAL-specific activity was considerably lower in the 4th week of the study compared to the controls.

**Figure 2:**
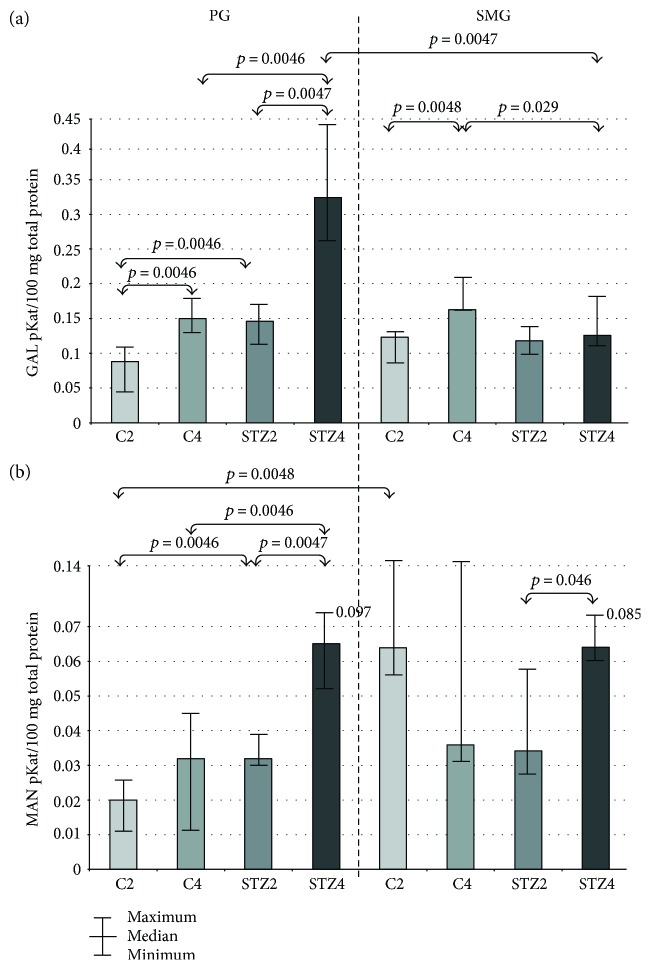
The effect of STZ-induced diabetes on activity of GAL and MAN in the parotid and submandibular glands of rats. C2 and C4 are the control groups. GAL: *β*-galactosidase; MAN: *α*-mannosidase; PG: parotid glands; SMG: submandibular glands; STZ2 and STZ4 are the diabetic groups.

We have shown that GAL-specific activity in parotid glands in the 4th week of the experiment was significantly higher compared to the submandibular glands (Figure 2(a)).

### 3.6. MAN

In parotid glands of rats with diabetes, MAN-specific activity in the 4th week of the experiment was significantly higher than in the 2nd week. We have also demonstrated that MAN-specific activity in parotid glands of rats with STZ-induced diabetes was considerably higher both in week 2 and 4 compared to the respective controls. In submandibular glands of diabetic rats, MAN-specific activity was significantly higher in the 4th week of the study compared to the 2nd week.

In week 2 of the experiment, MAN-specific activity in the control rats was considerably lower in parotid glands than in submandibular glands (Figure 2(b)).

### 3.7. FUC

**Figure 3:**
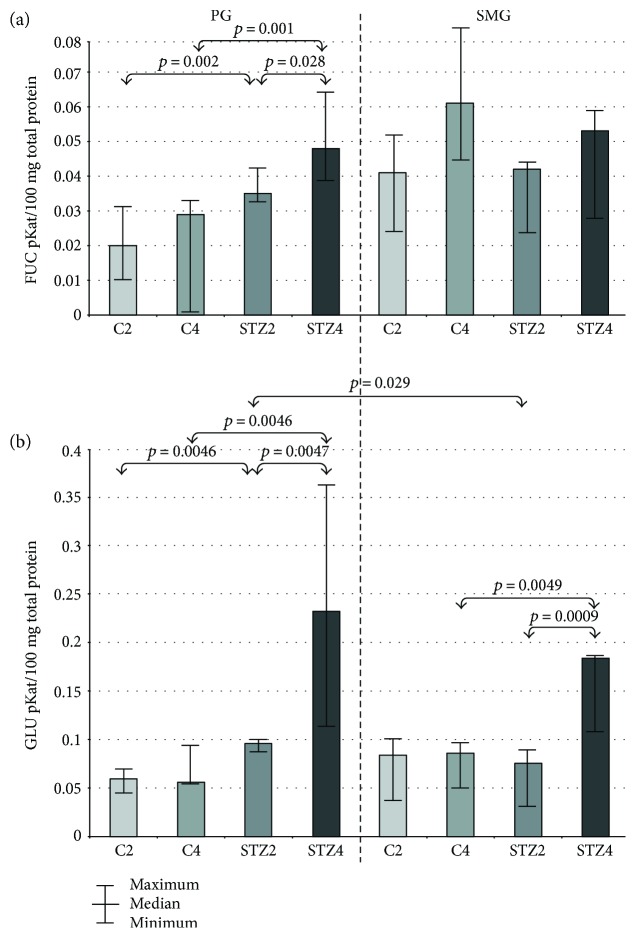
The effect of STZ-induced diabetes on activity of FUC and GLU in the parotid and submandibular glands of rats. C2 and C4 are the control groups; FUC: *α*-fucosidase (FUC); GLU: *β*-glucuronidase; PG: parotid glands; SMG: submandibular glands; STZ2 and STZ4: diabetic groups.

FUC-specific activity in parotid glands of rats with diabetes was significantly higher in week 4 of the experiment compared to week 2, and considerably higher in parotid glands of diabetic rats both in week 2 and 4 of the study compared to the control group. We did not observe any significant changes in FUC-specific activity in submandibular glands of the rats from both the control and study group (Figure 3(a)).

### 3.8. GLU

GLU-specific activity in parotid glands of rats with diabetes was significantly higher in the 4th week of the experiment compared to the 2nd week, and considerably higher in weeks 2 and 4 of the study in parotid glands of diabetic rats in comparison with the controls. In submandibular glands of STZ-induced diabetes rats, GLU-specific activity was significantly higher in week 4 of the experiment compared to week 2. In the 4th week of the study, the specific activity of GLU in diabetic rats was also considerably higher than in healthy controls.

In the 2nd week of the experiment, the group of rats with diabetes demonstrated significantly higher GLU-specific activity in parotid glands compared to submandibular glands (Figure 3(b)).

### 3.9. Salivary Amylase

In parotid glands of diabetic rats, specific activity of salivary amylase was significantly lower in week 4 of the experiment compared to week 2. In both week 2 and 4 of the study, salivary amylase-specific activity was considerably lower in parotid glands of diabetic rats compared to the controls.

**Figure 4:**
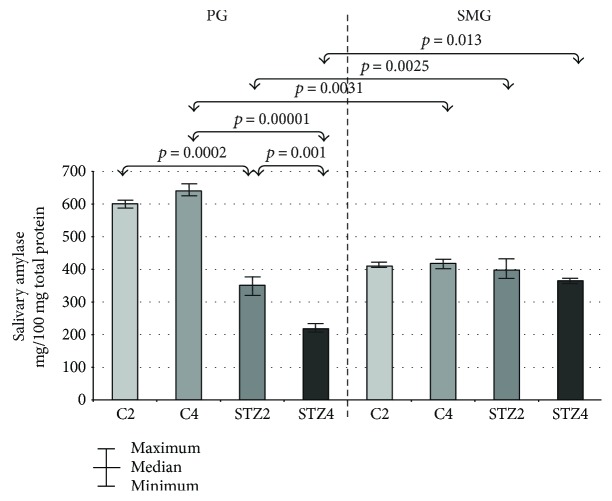
The effect of STZ-induced diabetes on salivary amylase activity in the parotid and submandibular glands of rats. C2 and C4 are the control groups; PG: parotid glands; SMG: submandibular glands; STZ2 and STZ4 are the diabetic groups.

In the control group, specific activity of salivary amylase in the 4th week of the study was significantly higher in parotid glands compared to submandibular glands, whereas in weeks 2 and 4 of the experiment, salivary amylase-specific activity in parotid glands of the diabetic rats was significantly lower compared to submandibular glands of these rats (Figure 4).

### 3.10. Correlations

In the 4th week of the experiment, there was positive correlation between the specific activity of HEX A and GAL in the parotid glands of rats with STZ-induced diabetes and blood glucose concentrations of these rats (*r* = 0.865, *p* = 0.001 and *r* = 0.729, *p* = 0.003). In week 4 of the study, in the parotid glands of STZ-diabetic rats, there was negative correlation between the specific activity of HEX B and stimulated salivary flow (*r* = −0.597, *p* = 0.01). In the 4th week of the experiment, in the parotid glands of STZ-diabetic rats, there was negative correlation between the specific activity of HEX B and specific activity of *α*-amylase (*r* = −0.745, *p* = 0.03). In the 2nd and 4th weeks of the experiment, there was negative correlation between the specific activity of salivary amylase in the parotid glands of rats with STZ-induced diabetes and the levels of blood glucose in these rats (*r* = −0.886, *p* = 0.019 and *r* = −0.829, *p* = 0.042, resp.). In week 4 of the experiment, in the parotid glands of diabetic rats, there was negative correlation between the specific activity of salivary amylase and the weight of the parotid glands (*r* = −0.929, *p* = 0.001).

### 3.11. Effect of STZ-Induced Diabetes on Histological Observation in the Salivary Glands

**Figure 5:**
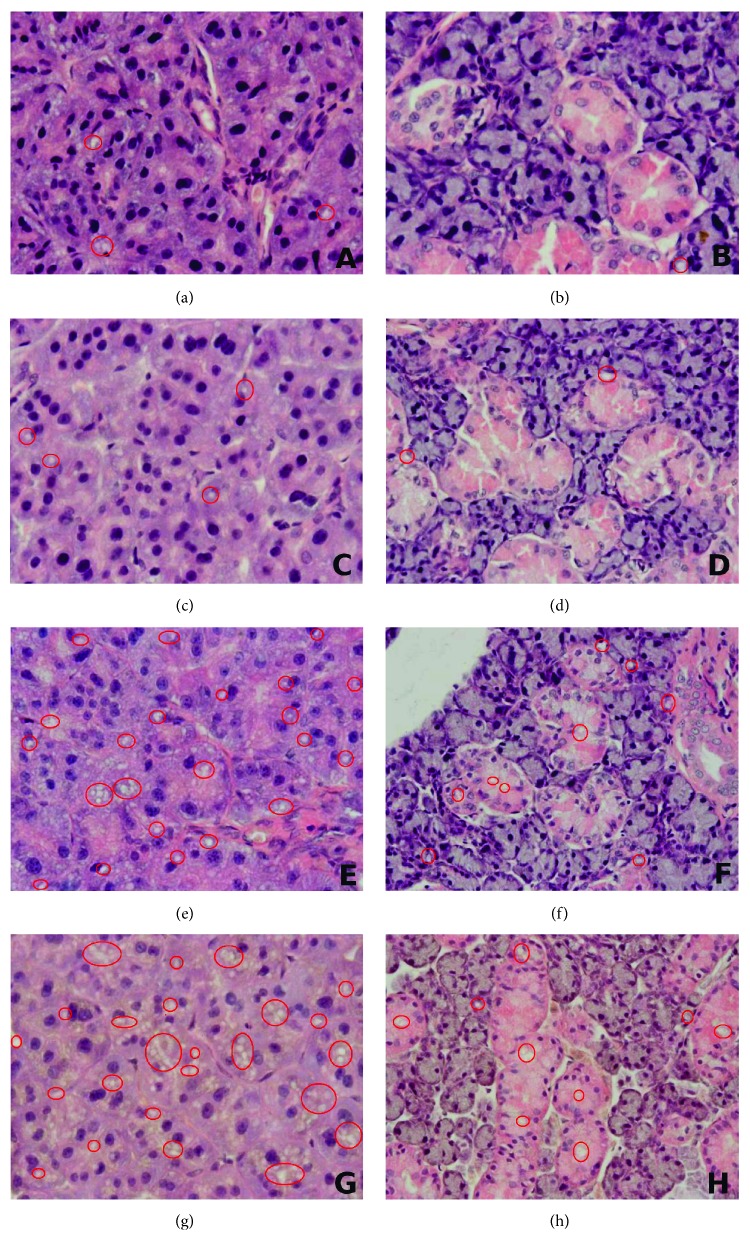
The effect of STZ-induced diabetes on histological observation in the salivary glands. C2 and C4 are the control groups; STZ2 and STZ4 are the diabetic groups; (a) parotid gland of the C2 rats; (b) submandibular gland of the C2 rats; (c) parotid gland of the C4 rats; (d) submandibular gland of the C4 rats; (e) parotid gland of the STZ2 rats; (f) submandibular gland of the STZ2 rats; (g) parotid gland of the STZ4 rats; (h) submandibular gland of the STZ4 rats; red circles indicate vacuoles.

In the submandibular and parotid glands of rats with STZ-induced diabetes, acinar cells showed a degenerative change in the form of vacuolation. In both the 2nd and 4th weeks of the experiment, the number of vacuoles was higher in the parotid gland as compared to the submandibular gland (Figure 5, Table 3).

## 4. Discussion

This is the first study that evaluated the relationship between lysosomal exoglycosidase profile and secretory function of the parotid and submandibular glands of rats with STZ-induced diabetes. The results of our paper demonstrated that experimental type 1 diabetes increases activity of most lysosomal exoglycosidases as well as impairs the salivary gland function observed as decrease of stimulated and nonstimulated salivary secretion, lowers the total protein content, and also reduced *α*-amylase activity.

An important group of lysosomal enzymes produced in the salivary glands is salivary lysosomal exoglycosidases. They hydrolyze a single monosaccharide from the nonreducing end of salivary glycoconjugates (e.g., glycoproteins and glycolipids) [31]. Since the activity of salivary exoglycosidases (especially HEX and GLU) correlates with the intensity of many diseases, it is suggested that these lysosomal hydrolases may be a potential noninvasive biomarker of many pathological conditions in the human body [11, 18, 32, 33].

In our study, we have shown that the activity of most salivary exoglycosidases is significantly higher in both parotid (GLU, GAL, MAN, and FUC in week 2 of the experiment; HEX A, GLU, GAL, MAN, and FUC in week 4) and submandibular diabetic glands (HEX and HEX A in week 2, GLU in week 4) as compared to the healthy controls. We have also demonstrated that the activity of salivary exoglycosidases in the parotid (HEX B, GLU, GAL, MAN, and FUC) and submandibular (HEX B, GLU, and MAN) diabetic glands varies considerably with the duration of the disease. The specific activity of lysosomal enzymes does not depend on the type of salivary glands in both good health and streptozotocin-induced diabetes.

HEX, the most active salivary exoglycosidase, is synthesized by mucosal and epithelial cells of salivary ducts, which is the main area of HEX synthesis in the salivary glands of rats [34]. The presented study showed that in the parotid glands of STZ-diabetic rats, HEX-specific activity was significantly higher in the 4th week of the experiment as compared to the 2nd week, while in the submandibular glands, it was statistically lower in the 4th week of the study. Higher HEX activity may indicate intensified tissue reconstruction in the parotid glands of rats with STZ-induced diabetes. In our study, also HEX B-specific activity was statistically higher in both submandibular (↑ 147%) and parotid glands (↑ 126%) of rats with STZ-induced diabetes (in the 4th week as compared to the 2nd week). Since the increase in HEX B activity may be considered as a marker of cellular membrane damage [24, 35, 36], these results suggest severe degenerative changes in both types of salivary glands along with the duration of STZ-induced diabetes. In the 4th week of the experiment as compared to the 2nd week, we also observed a significantly lower HEX A-specific activity in the submandibular glands as well as a considerable higher HEX A-specific activity in the parotid glands of rats with STZ-induced diabetes. These results may, respectively, indicate the lower and higher catabolism of negatively charged glycoconjugates depending on the rats' age and duration of experimental DM1. Higher HEX A activity is also considered to be a sensitive and early marker of diabetic microangiopathy, signalizing vascular endothelial dysfunction, and vascular organ injury [37, 38]. However, in the 4th week of the study, we demonstrated significantly lower HEX A-specific activity in diabetic submandibular glands versus control glands. This significant decline in HEX A activity may be explained by Romero et al. [39] who showed a decrease in the total sialic acid (TSA) in the saliva of rats with STZ-induced diabetes compared to the control group. The authors suggest that this may be due to reduced activity of enzymes involved in the synthesis of complex and negatively charged N-glycans (containing N-terminal sialic acid) [39]. Since acidic N-glycans are a substrate for HEX A activity [39], we may suppose that the depletion of this enzyme results from the substrate deficiency caused by disturbances in glycosylation processes. Our assumptions, however, require more detailed studies.

Changes in HEX A activity may correspond with the observed alternations in GAL activity. The specific activity of GAL was significantly lower in diabetic submandibular glands versus control glands in week 4 of the study. On the one hand, this may be a result of accumulation of salivary GAGs as well as hypertrophic changes in diabetic submandibular glands. On the other hand, it may be due for disturbances in salivary glycosylation suggested by Romero et al. [39]. Additionally, we have also reported a considerable increase in GAL activity (66% ↑ in week 2 and 116% ↑ in week 4) in diabetic parotid gland compared to control glands, which may prove enhanced hydrolysis of dermatan and keratan sulfates in the vascular wall of the parotid glands [40]. It is very likely that the observed changes in GAL and HEX A activities (both increase and decrease) may indicate functional anomalies in the blood vessel walls and other circulatory disorders in both types of salivary glands of rats with STZ-induced diabetes [40, 41]. It is considered that deterioration of salivary microcirculation reduces salivation and affects composition/buffering properties of saliva [3, 42]. These changes are typical for DM1 patients, which are characterized by microangiopathy and neuropathy as well as oral endothelial dysfunction [3, 43].

In our study, the salivary gland activity was assessed by measurement of nonstimulated and pilocarpine-stimulated salivary flow, *α*-amylase activity, and total protein concentrations. We have shown significantly lower *α*-amylase activity in the diabetic parotid glands relative to the control rats (in both week 2 and 4 of the experiment) as well as lower protein concentrations in the parotid glands of rats with STZ-diabetes (in 4 weeks of the study). *α*-Amylase is one of the key enzymes considered to be a specific marker for secretory function of salivary glands. Similarly to other salivary proteins, *α*-amylase is secreted into the saliva after stimulation of the sympathetic nervous system (SNS) [44, 45]. The results of our study suggest that STZ-induced diabetes is associated with impaired SNS neurotransmission, mainly in the parotid glands of rats. We hypothesize that this may be due to interrupted communication between the follicular cells of the salivary glands and nerve endings of SNS system [43, 44], resulting in a decrease of saliva production as well as reduced/impaired synthesis and/or secretion of salivary proteins. Synthesized proteins may have a faulty structure or be less functional or totally dysfunctional [2]. Decreased salivary secretion in STZ-diabetic group was also demonstrated by lower unstimulated (in the 4th week of the experiment) and stimulated (in the 2nd and 4th week of the experiment) salivary flow rate as compared to the healthy controls. Since stimulated salivary secretion reflects mainly the activity of the parotid gland [2], the results of our study showed that secretory function of this gland is primarily affected by STZ-induced diabetes. Particularly noteworthy is also a significant inverse correlation between the stimulated salivary flow rate, a specific activity of *α*-amylase, and HEX B in diabetic parotid gland in week 4 of the experiment, which may indicate the usefulness of HEX B for assessment of the parotid gland hypofunction in the course of STZ-induced diabetes.

The lower stimulated salivary secretion observed in this study might be a result of strong fat vacuolation (accumulation of fat) in the cells of parotid glands. Deconte et al. [46] reported massive accumulation of lipids in the parenchyma of follicular and mucosal cells, glandular tissue atrophy, and emergence of bands of the connective tissue as well as the accumulation of cells typical of chronic inflammation (lymphocytes, macrophages, and neutrophils) in the parotid and submandibular glands of STZ-diabetic rats. These changes, however, were particularly evident in the parotid glands and significantly less prominent in the submandibular glands of STZ-diabetic rats [46]. Indeed, our work has also proved greater intensity of degenerative changes in the parotid glands of STZ-diabetic rats (Table 3). Although the present study did not explain the nature of severe intracytoplasmic vacuolization in the parotid and submandibular glands (higher degree of vacuolization in the parotid glands), these vacuoles, however, appeared to be a lipid nature since they were removed during fixation and processing of the samples [26].

Polymorphonuclear leukocytes (PMNs), that is, neutrophils, eosinophils, and basophils, play a key role in the oral homeostasis participating in the production of antimicrobial peptides, proteolytic enzymes and reactive oxygen species (ROS) [47]. PMNs are also a rich source of GLU, which is a major marker of inflammation, influx, and activity of neutrophils [48]. In our work, GLU-specific activity was significantly higher in the parotid (in the 2nd and 4th week of the experiment) and submandibular glands (in the 4th week of the experiment) of the diabetic rats versus control rats, and it significantly increased in both types of glands of STZ-diabetic rats as the experiment progressed. Enhanced GLU activity in the parotid and submandibular glands may indicate the existence of inflammation in the experimental model of type 1 diabetes, which occurs at the early stage of disease and is more severe at the advanced DM stages in the parotid glands of rats. It is well known that metabolic abnormalities associated with DM1 lead to hyperplasia and hypertrophy of adipocytes, as well as stimulate the release of the monocyte chemoattractant protein-1 (MCP-1) by these cells [49, 50]. MCP-1 increases the influx of monocytes into adipose tissue and promotes changing them into macrophages [2, 50]. Macrophages, by the release of proinflammatory cytokines (e.g., TNF-*α*, IL-6, and IL-1*β*), aggravate inflammation [2], which may be explained by the increased GLU activity in the STZ-induced diabetes.

Less active salivary exoglycosidases, *α*-mannosidase (MAN), and *α*-fucosidase (FUC) also appear to play an important role in the oral environment. We have demonstrated that MAN-specific activity was significantly higher in both glands of DM1 rats throughout the entire study, wherein FUC-specific activity was increased only in the parotid glands between week 2 and 4 of the experiment. Elevated FUC activity indicates a more severe catabolism of complex N-glycans in the parotid glands of STZ-diabetic rats in comparison to the submandibular glands. We speculate that metabolic disturbances in STZ-diabetes, similarly to abnormalities in the course of alcoholism [17], may inhibit enzyme mannosidase II resulting in intensive synthesis of high-mannose glycoproteins and increased MAN activity in both diabetic types of glands along with duration of STZ-induced diabetes. Significantly higher MAN activity in diabetic parotid glands versus control glands may also suggest that the balance between salivary glucosyltransferases and glycohydrolases is disturbed in STZ-induced type 1 diabetes [51, 52]. Additionally, it may be assumed that the metabolic abnormalities associated with DM1 may lead to the inhibition of further glycosylation stages, involving the conversion of high-mannose glycoproteins into mature (complex and hybrid) glycoproteins as well as formation of the substantial amounts of high-mannose glycoproteins accelerating their degradation [11, 28]. This issue, however, requires more detailed studies.

Analyzing the results of the presented study, attention should also be paid to its limitations. In our experiment, we have used an animal model, which can never replace the human model. The streptozotocin dose, duration of the experiment, and evaluation of only the selected lysosomal exoglycosidases also constitutes an imperfection of the study.

## 5. Conclusions

1. STZ-induced diabetes increased the activity of most lysosomal exoglycosidases in the parotid and submandibular salivary glands.
2. The activity of most lysosomal exoglycosidases in both glands of diabetic rats varies considerably with duration of the disease.
3. STZ-induced diabetes leads to disruption of the functioning of the parotid glands, which is reflected by a significant decrease in the specific activity of salivary amylase, total protein content, and stimulated salivary flow rate.

## Figures and Tables

**Table 1:** The effect of STZ-induced diabetes on body weight, glycaemia, plasma insulin, and salivary gland weight [M (min–max)].

	C2 *n* = 8	C4 *n* = 8	STZ 2 *n* = 8	STZ 4 *n* = 8
Body weight (g)	278 (265–289)	335^#^ (301–345)	234^∗^ (215–256)	234^∗^ (201–256)
Glycaemia (mg/dL)	95 (91–97)	94 (91–97)	298^∗^ (274–302.7)	316.7^∗^^,#^ (301.7–340)
Insulin (mU/mL)	4.5 (3-4.7)	4.3 (2.9–4.6)	0.000	0.000
Parotid gland weigh (g)	0.064 (0.054–0.075)	0.09 (0.084–0.099)	0.06 (0.057–0.078)	0.051^∗^^,#^ (0.038–0.054)
Submandibular gland weight (g)	0.145 (0.115–0.177)	0.153 (0.15–0.155)	0.141 (0.102–0.159)	0.134 (0.93–0.144)

M: median; min: minimum; max: maximum; C2 and C4 are the control groups; STZ2 and STZ4 are the diabetic groups; STZ2 versus C2 or STZ4 versus C4: ^∗^*p* < 0.05; C4 versus C2 or STZ4 versus STZ2: ^#^*p* < 0.05.

**Table 2:** The effect of STZ-induced diabetes on salivary flow and total protein concentration [M (min–max)].

	C2 *n* = 8	C4 *n* = 8	STZ 2 *n* = 8	STZ 4 *n* = 8
Unstimulated flow rate (*μ*L/min)	0.35 (0.2–0.56)	0.36 (0.22–0.61)	0.32 (0.06–0.52)	0.27^∗^ (0.067–0.91)
Stimulated flow rate (*μ*L/min)	88.6 (75.9–130.2)	86.4 (70.2–109.8)	63.79^∗^ (59.94–78.7)	52.7^∗^ (48.56–69.24)
Total protein in the parotid glands (*μ*g/mL)	4635.8 (4050.9–4689.1)	3997.3 (3941.2–4500.8)	4648.05 (4105.4–4829.9)	2060.3^∗^ (1527.8–2589.4)
Total protein in the submandibular glands (*μ*g/mL)	4416.35 (4139.1–5009.5)	4527.3 (4052.1–5355.2)	4684.1 (3812.9–4775.1)	4913.35 (4562.6–5225.0)

M: median; min: minimum; max: maximum; C2 and C4 are the control groups; STZ2 and STZ4 are the diabetic groups; STZ2 versus C2 or STZ4 versus C4: ^∗^*p* < 0.05.

**Table 3:** The effect of STZ-induced diabetes on histological observation in the salivary glands.

	C2 *n* = 8	C4 *n* = 8	STZ 2 *n* = 8	STZ 4 *n* = 8
Submandibular glands	8 (+)	8 (+)	7 (++), 1 (+++)^∗^	7 (+++), 1 (++++)^∗^
Parotid glands	8 (+)	7 (+), 1 (++)	4 (+++), 4 (++++)^∗^^,#^	8 (+++++)^∗^^,#^

+: single vacuoles in the cytoplasm of acinar cells; ++: 5–10% of the each section occupied by pathological changes; +++: 11–20% of the each section occupied by pathological changes; ++++: 21–30% of the each section occupied by pathological changes; +++++: >30% of the each section occupied by pathological changes; C2 and C4 are the control groups; STZ2 and STZ4 are the diabetic groups; STZ2 versus C2 or STZ4 versus C4: ^∗^*p* < 0.05; parotid versus submandibular: ^#^*p* < 0.05.
